# The Ectomycorrhizal Fungal Community in a Neotropical Forest Dominated by the Endemic Dipterocarp *Pakaraimaea dipterocarpacea*

**DOI:** 10.1371/journal.pone.0055160

**Published:** 2013-01-31

**Authors:** Matthew E. Smith, Terry W. Henkel, Jessie K. Uehling, Alexander K. Fremier, H. David Clarke, Rytas Vilgalys

**Affiliations:** 1 Department of Plant Pathology, University of Florida, Gainesville, Florida, United States of America; 2 Department of Biological Sciences, Humboldt State University, Arcata, California, United States of America; 3 Department of Fish and Wildlife Resources, University of Idaho, Moscow, Idaho, United States of America; 4 Department of Biology, University of North Carolina Asheville, Asheville, North Carolina, United States of America; 5 Department of Biology, Duke University, Durham, North Carolina, United States of America; University of California, Berkeley, United States of America

## Abstract

Ectomycorrhizal (ECM) plants and fungi can be diverse and abundant in certain tropical ecosystems. For example, the primarily paleotropical ECM plant family Dipterocarpaceae is one of the most speciose and ecologically important tree families in Southeast Asia. *Pakaraimaea dipterocarpacea* is one of two species of dipterocarp known from the Neotropics, and is also the only known member of the monotypic Dipterocarpaceae subfamily Pakaraimoideae. This Guiana Shield endemic is only known from the sandstone highlands of Guyana and Venezuela. Despite its unique phylogenetic position and unusual geographical distribution, the ECM fungal associations of *P. dipterocarpacea* are understudied throughout the tree’s range. In December 2010 we sampled ECM fungi on roots of *P. dipterocarpacea* and the co-occurring ECM tree *Dicymbe jenmanii* (Fabaceae subfamily Caesalpinioideae) in the Upper Mazaruni River Basin of Guyana. Based on ITS rDNA sequencing we documented 52 ECM species from 11 independent fungal lineages. Due to the phylogenetic distance between the two host tree species, we hypothesized that *P. dipterocarpacea* would harbor unique ECM fungi not found on the roots of *D. jenmanii*. Although statistical tests suggested that several ECM fungal species did exhibit host preferences for either *P. dipterocarpacea* or *D. jenmanii*, most of the ECM fungi were multi-host generalists. We also detected several ECM fungi that have never been found in long-term studies of nearby rainforests dominated by other *Dicymbe* species. One particular mushroom-forming fungus appears to be unique and may represent a new ECM lineage of Agaricales that is endemic to the Neotropics.

## Introduction

Ectomycorrhizal (ECM) fungi are a diverse functional group of mutualistic root symbionts that enhance host plant nutrient acquisition, protect against root disease, and mitigate the effects of abiotic stresses [1], [2]. The ECM symbiosis was historically considered to be restricted to the temperate regions of the world where many forests are dominated by ECM plants. However, evidence has steadily accumulated over the last 50 years that ECM plants and fungi are present in most tropical ecosystems. Tropical ECM plants are most often present at low densities in plant communities dominated by arbuscular mycorrhizal plants, but at specific tropical sites ECM plants can be dominant components of the vegetation [3], [4], [5]. The recognition that ECM plants are widely distributed in the tropics has fostered a growing interest in their symbiotic ECM fungi. Because tropical habitats are often challenging to access, there are still major gaps in our understanding of the ecology, biogeography, and host preferences of tropical ECM fungi and plants. Several recent studies have suggested that tropical forests harbor limited ECM fungal diversity with either few or no endemic ECM fungal lineages [6]. In contrast, other tropical studies have detected relatively high ECM fungal diversity and presented evidence that at least some ECM fungal lineages originated from or diversified in the tropics [7], [8], [9].

In the Neotropics, several unrelated plant genera have independently evolved the ability to form ECM symbioses with fungi: *Pakaraimaea* (Dipterocarpaceae), *Quercus* (Fagaceae), *Coccoloba* (Polygonaceae), *Aldina* (Fabaceae subfamily Papilionoideae), *Dicymbe* (Fabaceae subfamily Caesalpinioideae), *Gnetum* (Gnetaceae) and at least three genera in the Nyctaginaeae (*Pisonia*, *Neea*, and *Guapira*) [4], [7], [9], [10], [11], [12]. These primarily lowland neotropical ECM plants are highly variable in terms of growth habit and geographic distribution. For example, species of Nyctaginaceae, *Coccoloba*, and *Gnetum* are shrubs, small trees, or lianas widely distributed at low densities in many forest types [10], [13] whereas species of *Quercus* and *Dicymbe* are canopy trees that tend to dominate stands but have more restricted geographic distributions [4], [7]. Lowland regional distributions of these ECM plant genera range from pantropical (*Gnetum*) to neotropical (*Coccoloba*) to Central American (*Quercus*) to Guiana Shield-endemic (*Dicymbe*, *Aldina*, *Pakaraimaea*). The ECM fungi associated with most of these plant genera have been characterized to various degrees, but to date the fungal symbionts of *Gnetum* and *Pakaraimaea* have been insufficiently studied.

The angiosperm family Dipterocarpaceae is one of the most ecologically and economically important tropical ECM plant lineages [8], [11], [14], [15]. This primarily Old World family contains more than 500 species and many are large, emergent trees that dominate forests in Southeast Asia and to a lesser extent in Africa [16]. The mycorrhizal biology of dipterocarps was recently reviewed by Brearley [17]. The family Dipterocarpaceae was considered restricted to the paleotropics until the latter 20^th^ century when the monotypic genera *Pakaraimaea* and *Pseudomonotes* were described from the Guiana Shield region of South America [18], [19]. *Pseudomonotes tropenbosii* Londoño, Alvarez & Forero (Dipterocarpaceae subfamily Monotoideae) is known only from southeastern Colombia [19] and was recently shown to be associated with sporocarps of putatively ECM fungi [20]. *Pakaraimaea* is represented by one species (*P. dipterocarpacea* Maguire & P. S. Ashton) with two subspecies (*nitidum* and *dipterocarpacea*), and forms dense stands of coppicing trees in savanna-fringing forests in the sandstone uplands of Guyana and Venezuela [18], [21]. *Pakaraimaea dipterocarpacea* is the only known member of Dipterocarpaceae subfamily Pakaraimoideae, an intermediate lineage between subfamilies Dipterocarpoideae and Monotoideae [22], [23].

Despite the unique phylogenetic position and unusual distribution of *P. dipterocarpacea*, relatively little is known about the ECM fungal communities associated with this tree. Moyersoen [11] studied roots of several individuals of *P. dipterocarpacea* ssp. *nitidum* in Venezuela and confirmed their association with seven species of ECM fungi. In a follow up study, Moyersoen [24] identified an additional 31 species of ECM fungi based on collections of ECM roots and sporocarps, but unfortunately molecular data are unavailable for many of these taxa. The ECM fungi associated with the Guyanese *P. dipterocarpacea* spp. *dipterocarpacea* have never been studied.

A recent study of ECM fungi in a mixed tropical rainforest in Ecuador found low fungal diversity but strong host preferences [10]. In contrast, we recently documented high ECM fungal diversity but no apparent fungal host preferences on the three co-occurring leguminous host trees *Dicymbe corymbosa* Spruce ex Benth., *Dicymbe altsonii* Sandw., and *Aldina insignis* (Benth.) Endl. in the Pakaraima Mountains of Guyana [9]. Further explorations in this region identified the nearby Pegaima savanna-forest mosaic as a habitat where the leguminous ECM tree, *Dicymbe jenmanii* Sandw., co-occurs with the dipterocarp *P. dipterocarpacea*. In portions of the savanna-fringing forest, *P. dipterocarpacea* grows as large, coppicing trees that dominate the canopy interspersed with medium-sized individuals of *D. jenmanii* that reach the mid- to upper-canopy. The co-occurrence of these two distantly related, Guiana Shield endemic, ECM-forming tree species in close proximity to our previous study sites provided a unique opportunity to further explore fungal host preferences and beta diversity of ECM fungi in this remote neotropical region.

For this study we sampled the ECM fungal communities of *P. dipterocarpacea* and *D. jenmanii* where these host plants co-occur in the Pegaima savanna. We asked the following questions: 1) Do ECM fungi exhibit marked host preferences for either one or the other plant species?, 2) Does *P. dipterocarpacea* host ECM fungal species not found on leguminous trees of the region? and, 3) Are the dominant ECM fungi in this dipterocarp-dominated forest different from those of the Fabaceae-dominated ECM communities in nearby rainforests?

## Methods

### Study Site and Host Plants

**Figure 1:**
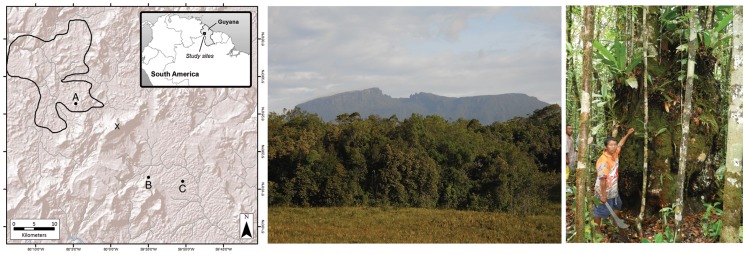
Location and appearance of forests dominated by *Pakaraimaea dipterocarpacea.* Map (left) showing the central Pakaraima Mountain region of western Guyana including the Upper Potaro and Mazaruni River Basins. The tallest mountain in Guyana, (Mt. Ayanganna, 2041 m) is indicated by an X. This study was conducted in *Pakaraimaea dipterocarpacea*-*Dicymbe jenmanii* stands at 800 m elevation at the edge of the Pegaima savanna, with location indicated by A. The approximate extent and location of the savanna ecosystem is shown with a black polygon; the rest of the map area is forested. The locations of other study plots in closed-canopy *Dicymbe* rainforests at ∼800 m elevation along the Potaro river are indicated by B (Henkel et al. [25]) and C (Smith et al. [9]). The center photo shows an ecotone of savanna with fringing forest dominated by *Pakaraimaea dipterocarpacea*, Pegaima savanna, Upper Mazaruni Basin, Guyana. The photo on the right shows an individual tree of *Pakaraimaea dipterocarpacea* measuring 212 cm diameter at breast height.

Fieldwork for this study was conducted during December 2010-January 2011 and May-June 2012 in the Upper Mazaruni River Basin in the Pakaraima Mountains of Guyana (Fig. 1). This site is located within a large complex of open savanna communities intermixed with patches of closed-canopy fringing forest on the western side of Mt. Ayanganna, the highest sandstone mountain in Guyana (2041 m). Previous observations indicated that the savanna-fringing forests were dominated by *P. dipterocarpacea* ssp. *dipterocarpacea* (hereafter *P. dipterocarpacea*) with *D. jenmanii* as a common subdominant, with a general upper canopy height of ∼20 m. We established a base camp at ∼800 m elevation at 5° 26′ 21.3′′ N; 60° 04′ 43.1″ W. This area is ca. 25 km from the rainforest sites on the eastern side of Mt. Ayanganna in the Upper Potaro River Basin where we have conducted multi-year sampling of ECM fungal sporocarps in *Dicymbe*-dominated forests [25] and belowground studies of ECM fungi with multiple leguminous host tree species [9]. The Potaro and Pegaima sites are geographically close but vary in annual precipitation (>2400 mm and ∼2000 mm, respectively); the Pegaima site is drier due to its position within the rain shadow of Mt. Ayanganna [26], [27]. Pegaima soils are highly oligotrophic white sands (entisols) derived from the Roraima Formation sandstone whereas the soils from the specific Potaro rainforest study sites are laterites derived from intrusive igneous rock (oxisols) [4], [28]. Burn scars on forest edge trees indicated that the Pegaima site experiences periodic anthropogenic or natural fires, whereas fires are extremely rare or absent from the Potaro rainforests [26], [29].

We initially examined fertile collections of *P. dipterocarpacea* to determine which of the two subspecies was present. Several publications have documented the distribution and growth habit of the two subspecies in the seasonally dry areas of the Pakaraima range of Guyana and throughout the Caroni River Basin of Venezuela [11] and references therein]. Plant identification was based on leaf and petal morphology; *P. dipterocarpacea* ssp. *dipterocarpacea* (Guyana) has shorter leaves and glabrous petals whereas ssp. *nitidum* (Venezuela) has longer leaves and pubescent petals [30]. Fertile plant voucher specimens were photographed in the field and deposited in the BRG (Guyana), US, and HSU herbaria. At the Pegaima site, *P. dipterocarpacea* dominates the fringing forests, composing >50 percent of the basal area and upper canopy area, with most individuals exhibiting multiple, co-dominant canopy stems and numerous sprout shoots of various sizes (Henkel, unpublished data). Previous reports suggested that *P. dipterocarpacea* is usually encountered as a small, shrubby tree and that large emergent individuals are rare [31]. However, at Pegaima individual trees of *P. dipterocarpacea* regularly reach 15–20 m in height and 100–200 cm diameter at breast height (dbh). Consistent with previous observations, the forest floor in stands dominated by *P. dipterocarpacea* at Pegaima were covered by a 10–20 cm thick litter layer [11].

### Ectomycorrhizal Root and Sporocarp Sampling

Root sampling followed protocols similar to Smith et al. [9] with minor changes. We identified 20 pairs of *P. dipterocarpacea* and *D. jenmanii* in which each tree was >20 cm dbh and where trees in a pair occurred ≤20 m from each other. A total of 40 trees (20 trees per species) were sampled along the edge of the Pegaima savanna in forests dominated by *P. dipterocarpacea* within ca. 0.3 km of base camp. Distances between sampled tree pairs ranged from 3–20 m (mean = 12.5 m). Four lateral roots from each sampled tree were traced 1–3 m from the base to the fine roots, where roots, soil, and litter were excavated and pooled until ca. 1000 cm^3^ of material rich in ECM roots was obtained. Roots were harvested only when unequivocally traced back to the sample tree. No molecular tests were used to verify the identity of the plant roots because our previous study showed that we could accurately determine hosts in the field as long as careful root tracing was conducted [9]. Root samples were rinsed in water to remove soil particles and inspected under a dissecting microscope. Eight ECM roots were randomly selected from each tree. In the study by Smith *et al.* [9] root morphotyping was used prior to molecular sampling but in this study no morphological sorting was conducted. A total of 320 individual ECM roots were rapidly dried in 8-strip microcentrifuge tubes by placing them overnight in a sealed container with silica gel.

Sporocarps of putative ECM fungi were collected at the Pegaima site in December 2010 and June 2012. These were then compared to a database of sporocarps collected at two sites in *Dicymbe*-dominated rainforest near the Potaro River during 2000–2010 [25]. These fungi were identified to species/morphospecies but most of the putatively new taxa known only from the Pegaima site have not yet had their ITS rDNA sequenced. For information on site, specimen identification, and herbaria accessions see Henkel *et al*. [9], [12].

### Molecular Protocols and Fungal Identification

Molecular protocols for sequencing of ECM fungi from roots and sporocarps followed those of Smith *et al.* [9]. Briefly, silica gel-dried ECM roots were rinsed in water to remove soil particles and then crushed with forceps in tubes containing 25 µl of extraction buffer from an Extract-N-Amp Plant kit (Sigma-Aldrich, St. Louis, MO, USA). Crushed roots were incubated at 96°C for 10 minutes and then mixed with 25 µl of dilution buffer. Sporocarp DNA was extracted using a CTAB protocol [32] or the Extract-N-Amp Plant kit (Sigma-Aldrich, St. Louis, MO, USA).

Fungal ITS rDNA was PCR-amplified with forward primer ITS1F in combination with reverse primers ITS4 or ITS4B. When amplification with these primers was unsuccessful, we used reverse primer ITS2 instead [32], [33], [34]. PCR protocols followed Smith *et al.* [9]. Amplicons were visualized on 1.5% agarose gels stained with SYBR Green I (Molecular Probes, Eugene, OR, USA). Amplicons were cleaned with EXO and SAP enzymes [35]. Sequencing was performed with the above primers using Big Dye Sequencing Kit v.3.1 (Applied Biosystems, Foster City, CA, USA). Sequences were edited with Sequencher v.4.1 (Gene Codes Inc., Ann Arbor, MI, USA).

Sporocarps were identified based on a combination of morphological features and rDNA sequences. Taxa detected only on roots were identified to genus and their uniqueness at the species level determined using blastN comparisons against our sporocarp and ECM root sequence database as well as GenBank. Internal transcribed spacer (ITS) sequences were considered to represent the same operational taxonomic unit (OTU), a proxy for species, if they differed by <3% across the ITS region [9]. Taxa were assigned to the phylogenetically defined ECM fungal lineages recognized by Tedersoo *et al*. [36]. These lineages constitute monophyletic groups of fungi that have independently evolved the ability to form ECM symbioses with plants. ECM sequences that did not fit into these groups, did not match well with known saprotrophic fungi, and were found on multiple healthy ECM roots were putatively considered representatives of new ECM lineages.

### Statistical Analyses

Two common measures of diversity, the Shannon-Wiener diversity index (H’) and the Simpson’s diversity index (1-D), were calculated for ECM fungi using PC-ORD 6 [37]. We constructed sampling curves to compare the level of ECM sampling and diversity at the Pegaima savanna and Potaro rainforest sites. PC-ORD 6 was used to generate sampling curves and standard deviations based on 500 bootstrap subsamples. The two sampling curves were then graphed using Microsoft Excel based on the number of roots sampled at each site (8 ECM roots per tree at Pegaima, 20 ECM roots per tree at Potaro). To test for statistically significant differences in frequency of individual ECM fungal species on the two different host plants we used the software package R to perform a Fischer’s exact test for each fungal species that occurred more than one time in the dataset. The Fisher exact test is a significance test used in the analysis of contingency tables with low sample sizes. PC-ORD 6 was also used for preliminary ordination analyses to examine the effects of host plants on ECM fungal communities.

### Molecular and Phylogenetic Analysis of the Unique Ectomycorrhizal Fungus Agaricales TH9235

At both the Potaro and Pegaima sites we collected a large, orange-colored, tricholomatoid mushroom, with the stipitate, pileate and gilled macromorphology typical of the basidiomycete order Agaricales (voucher TH9235 from the Potaro site, voucher TH9693 from the Pegaima site). This mushroom produced a white spore print and fruited directly on soil in close proximity to *P. dipterocarpacea* and *D. jenmanii* at Pegaima and with *Dicymbe* spp. at Potaro. The mushroom had white hyphal cords at the base that were usually attached to clusters of white ECM roots. The taxonomic affinities of this fungus have proved particularly difficult to determine based on morphology or ITS rDNA sequences. During the course of this study, we also detected Agaricales TH9235 on ECM roots of *Dicymbe jenmanii*. Because of the putatively unique position of this fungus within the Agaricales and its potential as a neotropical endemic taxon, we initiated a preliminary exploration of the phylogenetic relationships of Agaricales TH9235 by sequencing portions of three additional regions: 1) 18S rDNA, 2) 28S rDNA, and 3) mtLSU. We followed the same general protocols as outlined above except we used primers ssu1536 and ssu0817 for 18S [38], LROR and LR5 for 28S [33] and ML5 and ML6 for mtLSU [39]. Sequence data from these three genes was subjected to blastN analysis.

We also performed a phylogenetic analysis using the 28S rDNA region. This region was selected because sequence data are available for many Agaricales species and it has proved useful for resolving the phylogeny of many mushroom-forming fungi [40]. For this analysis, we included representatives of all Agaricales genera that were similar to Agaricales TH9235 based on blastN analysis as well as several other white-spored ECM fungi from unrelated Agaricales lineages [40]. The 28S rDNA alignment that was used for phylogenetic analysis included 88 species of Agaricales and was 1012 bp long after exclusion of ambiguously aligned DNA regions. To assess the relationships between Agaricales TH9235 and other members of the Agaricales, we performed a Maximum Likelihood analysis using the GTR+I+G model (generalized time reversible+invariant sites+Gamma distribution) with the software package GARLI [41]. Statistical support was assessed by conducting 250 bootstrap replicates.

## Results

### Ectomycorrhizal Fungal Diversity and Community Structure

A total of 160 ECM roots were randomly selected for analysis from each of the two tree species for a total of 320 ECM tips. Of these, DNA sequences of ECM fungi were successfully produced from 125 ECM tips of *D. jenmanii* and 130 ECM tips of *P. dipterocarpacea* (78% and 81% success rates, respectively). A total of 52 ITS species-level operational taxonomic units (OTUs) of ECM fungi were recovered from the roots of *P. dipterocarpacea* and *D. jenmanii* (Table 1). Both the Shannon-Wiener Index (H’) diversity value for the total species assemblage and the Simpson’s Diversity Index value of 1-D were moderately high (1.434 and 0.7446, respectively) but slightly lower than the diversity index values found in nearby rainforest sites [9]. Seventeen frequently occurring OTUs were detected three or more times whereas 10 OTUs were found twice and 25 OTUs were detected only once. These ECM taxa represented 11 independent fungal lineages, with the majority occurring in the familiar/boletus,/russula-lactarius,/clavulina, and/tomentella-thelephora lineages (Table 1). Two OTUs (Polyporales ECM32_7 and Agaricales TH9235) did not fall into any of the known ECM lineages outlined by Tedersoo et al. [36]. Polyporales ECM32_7 was highly similar to a polyporoid OTU detected in Guyana on healthy ECM roots with a fungal mantle and Hartig net by Smith *et al.* [9] and is therefore considered an ECM fungus. The other unknown OTU found on ECM roots (Agaricales TH9235) matched tricholomatoid mushrooms collected at both the Pegaima and Potaro sites. This species putatively represents a new ECM lineage (see below).

**Table 1:** Ectomycorrhizal fungi detected on the roots of *Pakaraimaea dipterocarpacea* and *Dicymbe jenmanii* in this study.

ECM Taxon (OTU)	Sporocarp Voucher	ECM Lineage	GenBank Number	*Pakaraimaea dipterocarpacea*	*Dicymbe jenmanii*	Total	Found Previously?
***Boletellus ananas***	**TH9188**	**/boletus**	**JN168685**	**9**	**11**	**20**	**yes**
***Russula*** ** TH9503**	**TH9503**	**/russula-lactarius**	**KC155378**	**14**	**4**	**18**	**no**
***Xerocomus amazonicus***	**TH8839**	**/boletus**	**JN168782**	**10**	**4**	**14**	**yes**
***Austroboletus rostrupii***	**TH8189**	**/boletus**	**JN168683**	**9**	**3**	**12**	**yes**
***Tylopilus potamogeton v. irengensis***	**TH8801**	**/boletus**	**JN168779**	**4**	**5**	**9**	**yes**
***Amanita calochroa***	**MCA3927**	**/amanita**	**KC155375**	**4**	**4**	**8**	**yes**
***Russula*** ** TH9145**	**TH9145**	**/russula-lactarius**	**JN168752**	**3**	**5**	**8**	**yes**
***Cortinarius*** ** MCA3928**	**MCA3928**	**/cortinarius**	**JN168712**	**1**	**7**	**8**	**yes**
***Tomentella*** ** ECM1111**	**–**	**/tomentella-thelephora**	**JN168760**	**0**	**6**	**6**	**yes**
***Tomentella*** ** ECM40-5**	**–**	**/tomentella-thelephora**	**KC155370**	**0**	**6**	**6**	**no**
***Xerocomus*** ** ECM1082**	**–**	**/boletus**	**JN168783**	**3**	**2**	**5**	**yes**
***Cortinarius*** ** ECM953**	**–**	**/cortinarius**	**JN168710**	**1**	**3**	**4**	**yes**
***Cortinarius*** ** TH8613**	**TH8613**	**/cortinarius**	**KC155377**	**2**	**1**	**3**	**yes**
***Lactarius*** ** cf. ** ***annulifer***	**TH9014**	**/russula-lactarius**	**KC155376**	**2**	**1**	**3**	**yes**
***Russula*** ** ECM1056**	**–**	**/russula-lactarius**	**JN168740**	**1**	**2**	**3**	**yes**
***Cortinarius*** ** ECM34-5**	**–**	**/cortinarius**	**KC155361**	**0**	**3**	**3**	**no**
***Tomentella*** ** ECM34-4**	**–**	**/tomentella-thelephora**	**KC155372**	**0**	**3**	**3**	**no**
***Cortinarius*** ** TH8546**	**TH8546**	**/cortinarius**	**JN168714**	**2**	**0**	**2**	**yes**
***Lactarius*** ** TH9522**	**TH9522**	**/russula-lactarius**	**KC155399**	**1**	**1**	**2**	**no**
***Russula campinensis*** group **ECM21-7**	**–**	**/russula-lactarius**	**KC155369**	**1**	**1**	**2**	**no**
***Russula*** ** MCA1856**	**MCA1856**	**/russula-lactarius**	**JN168745**	**1**	**1**	**2**	**yes**
***Agaricales*** ** TH9235**	**TH9235**	**/agaricalesTH9235**	**KC155374**	**0**	**2**	**2**	**yes**
***Lactarius*** ** ECM1066**	**–**	**/russula-lactarius**	**JN168729**	**0**	**2**	**2**	**yes**
**Boletoid sequestrate sp. 2 TH9514**	**TH9514**	**/boletus**	**KC155381**	**0**	**2**	**2**	**no**
***Polyporales*** ** ECM32-7**	**–**	**/polyporales1**	**KC155368**	**0**	**2**	**2**	**no**
***Tomentella*** ** ECM755**	**–**	**/tomentella-thelephora**	**JN168765**	**0**	**2**	**2**	**yes**
***Tylopilus pakaraimensis***	**TH8965**	**/boletus**	**JN168778**	**0**	**2**	**2**	**yes**
***Boletaceae*** ** ECM9-7**	**–**	**/boletus**	**KC155365**	**1**	**0**	**1**	**no**
***Clavulina*** ** ECM31-6**	**–**	**/clavulina**	**KC155362**	**1**	**0**	**1**	**yes**
***Clavulina*** ** ECM972**	**–**	**/clavulina**	**JN168704**	**1**	**0**	**1**	**yes**
***Clavulina*** ** ECM1037**	**–**	**/clavulina**	**JN168692**	**1**	**0**	**1**	**yes**
***Clavulina*** ** ECM1089**	**–**	**/clavulina**	**JN168695**	**1**	**0**	**1**	**yes**
***Clavulina sprucei*** ** group - species 1**	**TH9122**	**/clavulina**	**HQ680355**	**1**	**0**	**1**	**yes**
***Elaphomyces*** ** ECM1108**	**–**	**/elaphomyces**	**JN168718**	**1**	**0**	**1**	**yes**
***Boletus*** ** ECM11-6**	**–**	**/boletus**	**KC155363**	**1**	**0**	**1**	**no**
***Cortinarius*** ** ECM37-1**	**–**	**/cortinarius**	**KC155366**	**1**	**0**	**1**	**no**
***Hysterangiales*** ** ECM25-4**	**–**	**/hysterangium**	**KC155367**	**1**	**0**	**1**	**no**
***Tomentella*** ** ECM15-5**	**–**	**/tomentella-thelephora**	**KC155371**	**1**	**0**	**1**	**no**
***Amanita*** ** TH8931**	**TH8931**	**/amanita**	**JN168680**	**0**	**1**	**1**	**yes**
***Boletellus dicymbophilus***	**TH8616**	**/boletus**	**KC155373**	**0**	**1**	**1**	**yes**
***Clavulina*** ** ECM1129**	**–**	**/clavulina**	**JN168698**	**0**	**1**	**1**	**yes**
***Clavulina*** ** ECM26-7**	**–**	**/clavulina**	**KC155364**	**0**	**1**	**1**	**no**
***Clavulina sprucei*** ** group - species 2**	**TH9586**	**/clavulina**	**JN247429**	**0**	**1**	**1**	**yes**
***Coltricia*** ** ECM731**	**–**	**/coltricia**	**JN168708**	**0**	**1**	**1**	**yes**
***Lactarius multiceps***	**TH9154a**	**/russula-lactarius**	**JN168731**	**0**	**1**	**1**	**yes**
*Russula* ECM36-2	–	/russula-lactarius	**KC238673**	1	0	1	no
***Russula campinensis*** ** group TH7403**	**TH7403**	**/russula-lactarius**	**JN168738**	**0**	**1**	**1**	**yes**
*Tomentella* ECM10-8	–	/tomentella-thelephora	**KC238674**	0	1	1	no
*Tomentella* ECM12-6	–	/tomentella-thelephora	**KC238675**	0	1	1	no
***Tylopilus vinaceipallidus***	**TH8859**	**/boletus**	**JN168780**	**0**	**1**	**1**	**yes**
***Xerocomus*** ** ECM1039**	**–**	**/boletus**	**JN168781**	**0**	**1**	**1**	**yes**
***Xerocomus*** ** TH8865**	**TH8865**	**/boletus**	**JN021114**	**0**	**1**	**1**	**yes**

Species-level operational taxonomic units (OTUs) are defined as sequences that are ≥97% similar across the ITS rDNA sequence region. Taxa labeled with Latin binomials or voucher numbers (TH, MCA) were identified based on ITS matches with sporocarps. Species with ECM numbers are known only from sequences obtained from ECM roots. All species are assigned to the ECM lineages defined in Tedersoo et al. [36]. The numbers shown in the columns labeled *Pakaraimaea dipterocarpacea* and *Dicymbe jenmanii* designate the number of occurrences of each fungal OTU per host species. In cases where a particular fungal OTU was detected on more than one root tip from an individual tree this was not counted as a separate occurrence. The column on the far right indicates whether or not an OTU has been found previously on ECM roots or as sporocarps at other sites in Guyana.

**Figure 2:**
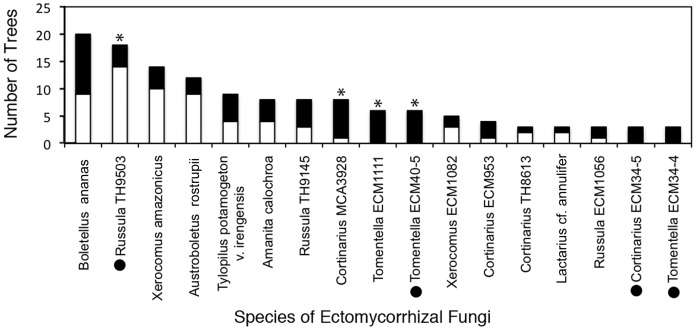
Frequency of occurrence of the 17 most common ectomycorrhizal (ECM) fungi on the roots of host trees *Pakaraimaea dipterocarpacea* (white bars) and *Dicymbe jenmanii* (black bars) at the Pegaima savanna site, Upper Mazaruni Basin, Guyana. Each of these common fungal species occurred on three or more individual trees; 20 trees were sampled for each of the host tree species. Species that showed a significantly different distribution on the two host plants (as assessed by Fischer’s Exact test) are indicated by asterisks. Fungal species that have never been found in previous ECM sporocarp or root surveys in nearby rainforest sites are designated by black circles. All other ECM fungal species have been found previously in association with species of *Dicymbe* and *Aldina* at other locations. Named fungal species are indicated by a genus and species binomial whereas species with TH or MCA numbers were matched to voucher specimens of undescribed species identified to genus. The ECM numbers correspond to fungal species known only from ECM root sequences.

Overall, 40 ECM fungal OTUs were detected on roots of *D. jenmanii* and 28 OTUs on *P. dipterocarpacea*. Individual trees of *D. jenmanii* hosted 2–7 species (mean 5 species per tree) and individual trees of *P. dipterocarpacea* hosted 2–7 species (mean 4 species per tree). Sixteen ECM OTUs were shared by both tree species. While 24 ECM fungi were unique to *D. jenmanii*, and 13 to *P. dipterocarpacea*, the majority of these were detected from a single root tip. Most of the common ECM fungi (13/17) were detected on the roots of both *P. dipterocarpacea* and *D. jenmanii* and 6 of the 8 most frequently detected species had a similar number of occurrences on the two host plants (Fig. 2). Four of the 17 common ECM fungi (*Cortinarius* ECM 34-5 and *Tomentella* ECM 40-5, *Tomentella* ECM 34-4 and *Tomentella* ECM1111) were only found on the roots of *D. jenmanii* whereas no ECM fungi were restricted to the host plant *P. dipterocarpacea*. Fisher’s exact tests determined that four species (*Russula* TH9503, *Cortinarius* MCA3928, *Tomentella* ECM1111, and *Tomentella* ECM40-5) showed a statistically skewed distribution on one of the two host plants. *Russula* TH9503 was more common on the roots of *P. dipterocarpacea* but the other three taxa were more common on *D. jenmanii*. Preliminary ordination analyses did not show any strong patterns of host-specificity at the community level and are thus not discussed further (data not shown).

**Figure 3:**
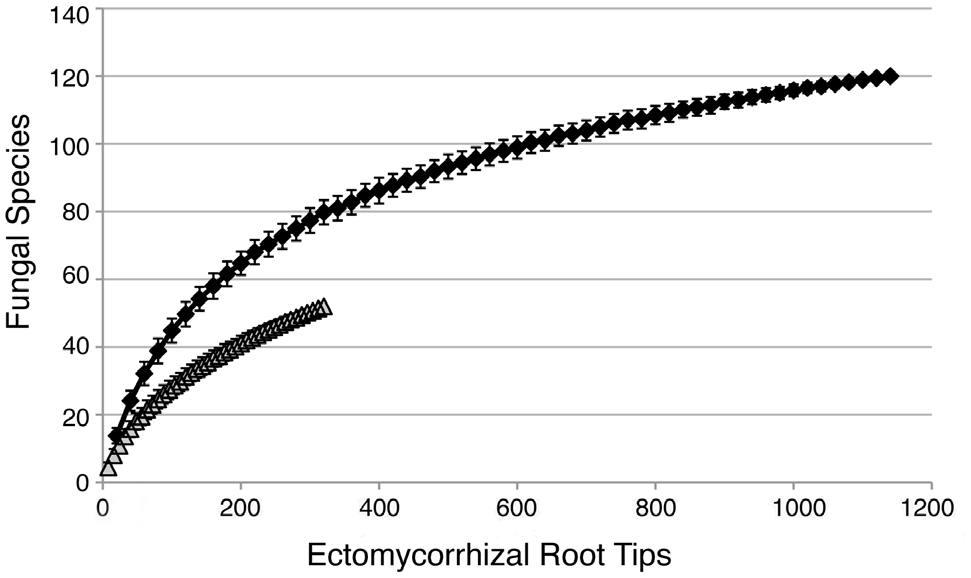
The sampling curves indicate the ectomycorrhizal (ECM) root tips sampled (X-axis) and number of ECM fungal species recovered (Y-axis) from the Pegaima site with *Pakaraimaea dipterocarpacea* and *Dicymbe jenmanii* (grey triangles, this study) and from the Potaro site with *Dicymbe corymbosa*, *Dicymbe altsonii*, and *Aldina insignis* (black squares, Smith et al. [**9**] **).** The two studies differed in their sampling procedure; this study was based on random sampling of eight ECM roots per tree whereas the study by Smith et al. [9] was based on sampling of 20 morphotyped ECM roots per tree.

Fourteen of the 52 fungal OTUs documented on ECM roots corresponded with formally described species and 24 of the 52 OTUs could be matched with vouchered sporocarp specimens from the Pegaima site. Approximately 67% of the fungi detected on ECM roots in this study (35/52 OTUs) have also been detected previously at other Guyanese rainforest sites dominated by leguminous trees, either directly as sequences on ECM roots or as sporocarps in long term research plots [9], [25]. Seventeen of the 52 OTUs detected on roots from the Pegaima site have not previously been found in Guyana on ECM roots or as sporocarps (Henkel & Smith, unpublished data). However, most of the common ECM species detected were fungi that have been found previously at other sites; among the 17 most common ECM fungal species at Pegaima, only four had not been detected before (*Russula* TH9503, *Tomentella* ECM40-5, *Tomentella* ECM34-4 and *Cortinarius* ECM34-5). Sampling effort curves indicated that while overall ECM fungal diversity was lower at the Pegaima site as compared with the Upper Potaro site of Smith et al. [9], much more sampling was needed at the Pegaima site to recover the total diversity of ECM fungi (Fig. 3).

Incomplete recovery of ECM fungal diversity by root sampling was corroborated by the fact that 82 species of putative or confirmed ECM fungi have now been collected as sporocarps from the same *P. dipterocarpacea*-*D. jenmanii* stands at Pegaima (Table 2). While the majority of these taxa are conspecific with species described or awaiting description from the Potaro sites [25], 26 are currently known only from the Pegaima sites, including species of *Russula*, *Lactarius*, *Clavulina*, *Cortinarius*, *Elaphomyces*, and *Sarcodon*. Taxonomic work on these potentially new species is ongoing.

**Table 2:** Ectomycorrhizal fungal taxa recovered as sporocarps in savanna-fringing forests dominated by host trees *Pakaraimaea dipterocarpacea* and *Dicymbe jenmanii* at the Pegaima site in the Upper Mazaruni Basin during 2011–2012.

Species^1^	ECM Lineage^2^	Pegaima Savanna Specimens^3^	Potaro Rainforest Specimens^3^	GenBank # (ITS)^4^
*Agaricales* TH9235	/agaricales TH9235	TH9693	TH 9235	KC155374,KC162210
*Amanita aurantiobrunnea* Simmons, T.W. Henkel & Bas	/amanita	TH 9685	TH 8937, MCA 3948	–
*Amanita calochroa* Simmons, T.W. Henkel & Bas	/amanita	TH 9662	MCA 3927	KC155375
*Amanita campinaranae* Bas	/amanita	TH 9552, 9700	TH 8453	KC155383
*Amanita craseoderma* Bas	/amanita	JKU 102	TH 8907	KC155382
***Amanita*** ** sp. 1**	/amanita	TH 9512	–	–
***Amanita*** ** sp. 2**	/amanita	JKU 95	–	–
*Amanita* sp. 3	/amanita	TH 9563, 9674	TH 8931	JN168680
*Amanita xerocybe Bas*	/amanita	TH 9663	TH 8930	KC155384
*Austroboletus rostrupii* (Syd. & P. Syd.) Horak	/boletus	TH 9508	TH 8189	JN168683
*Boletellus ananas* var. *ananas* (M.A. Curtis) Murrill	/boletus	TH 9500, 9668	TH 6264	JN168685
*Boletellus dicymbophilus* Fulgenzi & T.W. Henkel	/boletus	TH 9502, 9659, 9680	TH 8616	KC155373
*Boletellus exiguus* T.W. Henkel & Fulgenzi	/boletus	TH 9687	TH 9189	JN168687
boletoid sequestrate sp. 1	/boletus	TH 9555, 9661, 9689	TH 9163	JN168684
**boletoid sequestrate sp. 2**	/boletus	TH 9514, 9670	–	KC155381
*Cantharellus atratus* Corner	/cantharellus	TH 9679	TH 9203	JQ915107
*Clavulina* cf. *cinereoglebosa* Uehling, T.W. Henkel & Aime	/clavulina	JKU 100	TH 8561	JN228217
*Clavulina cirrhata* (Berk.) Corner	/clavulina	TH 9504, 9551	TH 8940	JQ677059
*Clavulina craterelloides* Thacker & T.W. Henkel	/clavulina	TH 9669	TH 8234	JQ911749
*Clavulina dicymbetorum* T.W. Henkel, Meszaros & Aime	/clavulina	TH 9533	TH 8730	DQ056364
*Clavulina humicola* T.W. Henkel, Meszaros & Aime	/clavulina	JKU 112	TH 8737	DQ056368
*Clavulina kunmudlutsa* T.W. Henkel & Aime	/clavulina	TH 9525, JKU 91	TH 8932	HQ680358
***Clavulina*** ** sp. 1**	/clavulina	TH 9679	–	–
***Clavulina*** ** sp. 2**	/clavulina	JKU 114	–	–
***Clavulina*** ** sp. 3**	/clavulina	JKU 93	–	–
***Clavulina*** ** sp. 4**	/clavulina	JKU 120	–	–
***Clavulina*** ** sp. 5**	/clavulina	JKU 121	–	–
*Clavulina sprucei* (Berk.) Corner^5^	/clavulina	TH 9528, 9567	TH 8221, 9122	HQ680354, HQ680355
*Coltricia* aff. oblectabilis	/coltricia	TH 9501, JKU 99	TH 9187	KC155387
*Coltricia* aff. navispora	/coltricia	TH 9516	MCA 3927	KC155386
*Coltricia* aff. montagnei	/coltricia	TH 9529, 9534	TH 9108	KC155388
***Coltricia*** ** sp. 4**	/coltricia	TH JKU 106	–	–
*Cortinarius* aff. *amazonicus* Singer & Araujo	/cortinarius	JKU 117	MCA 3928	JN168712
*Cortinarius* aff. *galeriniformis* Singer - species 1	/cortinarius	TH 9573, JKU 98	TH 8546	JN168714
***Cortinarius*** ** aff. ** ***galeriniformis*** ** Singer - species 2**	/cortinarius	TH 9520, 9532, 9686	–	–
*Cortinarius* aff. *kerrii* Singer	/cortinarius	TH 9543, 9686	TH 8539	KC155389
*Cortinarius* sp. 3	/cortinarius	TH 9510, 9518	TH 9178, MCA 3969	JN168713
***Cortinarius*** ** sp. 4**	/cortinarius	TH 9574	–	–
*Cortinarius* sp. 5	/cortinarius	TH 9511, 9683	TH 8613	KC155377
*Craterellus excelsus* T.W. Henkel & Aime	/cantharellus	TH 9527, 9530	TH 8235	JQ915102
*Craterellus olivaceoluteus* ined.	/cantharellus	TH 9539, 9656, 9665	TH 9205	JQ915109
*Craterellus pleurotoides* (T.W. Henkel et al.) A.W. Wilson	/cantharellus	TH 9526, 9703	TH 9220	JQ915110
*Craterellus cinereofimbriatus* ined.	/cantharellus	TH 9664	TH 8999	JQ915104
*Elaphomyces compleximuris* Castellano & S.L. Mill.	/elaphomyces	TH 9681	TH 8880	JN711441
*Elaphomyces digitatus* Castellano, T.W. Henkel & S.L. Mill.	/elaphomyces	TH 9535	TH 8887	JQ657705
***Elaphomyces*** ** sp. 1**	/elaphomyces	TH 9660	TH 9660	–
*Hysterangium* sp. 1^5^	/hysterangium	TH 9566, 9698	TH8517, MCA972	KC155391, KC155392
*Inocybe* cf. *pulchella* Matheny, Aime & T.W. Henkel	/inocybe	TH 9666	TH 9185	JN168726
***Inocybe*** ** sp. 1**	/inocybe	TH 9688	TH 9688	–
*Lactarius lignyophilus* ined.	/russula-lactarius	TH 9672	TH 7578	KC155398
***Lactarius*** ** sp. 1**	/russula-lactarius	TH 9558	–	–
***Lactarius*** ** sp. 2**	/russula-lactarius	TH 9522	–	KC155399
***Lactarius*** ** sp. 3**	/russula-lactarius	TH 9671	–	–
***Lactarius*** ** sp. 4**	/russula-lactarius	JKU 119	–	–
*Lactarius* sp. 5	/russula-lactarius	JKU 115	TH 7481	KC155400
*Pseudotulostoma volvata* O.K. Mill. & T.W. Henkel	/elaphomyces	JKU 103	TH 8975	JN168735
*Pulveroboletus* cf. *rosemariae* Singer	/boletus	TH 9571	TH 8232	JN168736
*Russula* aff. *puiggarii* (Speg.) Singer	/russula-lactarius	TH 9702	MCA 3954	JN168746
*Russula campinensis* (Singer) T.W. Henkel, Aime & S.L. Mill.	/russula-lactarius	TH 9556, JKU 118	TH 6844, 7403	JN168738
*Russula* cf. *leguminosarum* Singer	/russula-lactarius	TH 9547, JKU 110	TH 7425	KC155394
*Russula glutinovelata* S.L. Mill. & T.W. Henkel	/russula-lactarius	TH 9515, 9548, JKU 116	TH 8699	KC155395
*Russula metachromatica* ssp. *tarumaensis* Singer	/russula-lactarius	TH 9564	TH 7439	KC155393
*Russula myrmecobroma* S.L. Mill. & T.W. Henkel	/russula-lactarius	TH 9523, 9546	TH 9145	JN168752
***Russula*** ** sp. 1**	/russula-lactarius	TH 9572	–	–
***Russula*** ** sp. 2**	/russula-lactarius	TH 9503, 9667, JKU 108	–	KC155378
***Russula*** ** sp. 3**	/russula-lactarius	TH 9541		
***Russula*** ** sp. 4**	/russula-lactarius	TH 9542		
***Russula*** ** sp. 5**	/russula-lactarius	TH 9568	–	KC155397
***Russula*** ** sp. 6**	/russula-lactarius	TH 9673	–	–
***Russula*** ** sp. 7**	/russula-lactarius	TH 9676	–	–
***Russula*** ** sp. 8**	/russula-lactarius	TH 9695	–	–
***Sarcodon pakaraimensis*** ** ined.**	/phellodon-bankera	TH 9513	–	KC155390
*Tomentella* sp. 1	/tomentella-thelephora	TH 9557	TH 8977	JN168773
*Tomentella* sp. 2	/tomentella-thelephora	TH 9569	–	KC155401
*Tylopilus ballouii* (Peck) Singer	/boletus	TH 9694	TH 8916	JN168775
*Tylopilus exiguus* T.W. Henkel	/boletus	TH 9549, 9658	TH 8929	JN168776
*Tylopilus pakaraimensis* T.W. Henkel	/boletus	TH 9538	TH 8965	JN168778
*Tylopilus potamogeton* var. *irengensis* T.W. Henkel	/boletus	TH 9507	TH 8801	JN168779
*Tylopilus rufonigricans* T.W. Henkel	/boletus	TH 9704	TH 8925	KC155380
*Xerocomus amazonicus* Singer	/boletus	TH 9505, 9531, 9659	TH 8839	JN168782
*Xerocomus* sp. 1	/boletus	TH 9506, 9570, 9701	TH 8846, 8848	KC155379
*Xerocomus* sp. 2	/boletus	TH 9585	TH 9604	–

A total of 82 fungal species were found as sporocarps and 26 of these (bold) have not been collected from other study sites in Guyana. For comparison, the voucher numbers are shown for those taxa that have been found at nearby Potaro rainforest sites. GenBank numbers for ITS ribosomal DNA sequences are given for species where available.

1 Taxa lacking epithets are morphologically distinct but as yet unidentified to the species level; taxa with epithets followed by “ined.” have been tentatively determined as new to science but are yet to be formally described.

2 ECM lineages as identified by Tedersoo et al. (2010) except for the/agaricalesTH9235 lineage which is documented here for the first time.

3 Vouchers with TH (Terry Henkel) and JKU (Jessie K. Uehling) numbers are housed at Humboldt State University whereas MCA (M. Cathie Aime) numbers are housed at Purdue University.

4 GenBank numbers refer to specimens collected at the Potaro rainforest sites (see Smith et al., 2011 and Henkel et al. 2012) except in cases where a given species is only known from the Pegaima site.

5 Known to be a complex of cryptic species.

### Affinities of the Unique Species Agaricales TH9235

**Figure 4:**
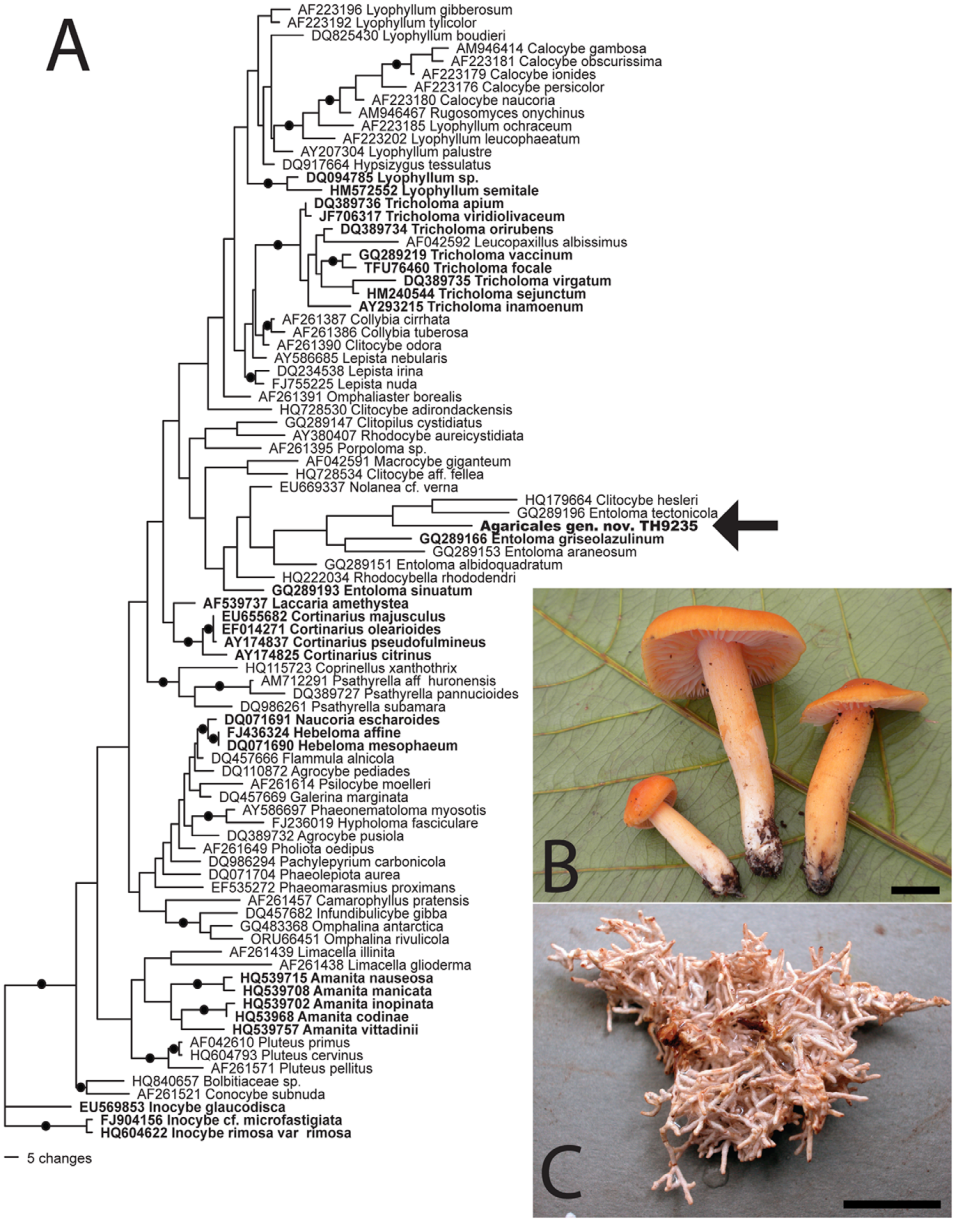
Phylogeny, morphology, and ecology of Agaricales TH9235. Maximum likelihood phylogeny (A) based on 28S rDNA shows inconclusive placement of Agaricales TH9235 within the mushroom-forming fungal order Agaricales (Basidiomycota). Nodes with bootstrap support ≥70 are indicated by black circles. Taxa considered ectomycorrhizal (ECM) based on Tedersoo et al. [36] are indicated by bold text, all other species are considered to be either saprotrophic, parasitic, or have an unknown trophic mode. Agaricales TH9235 is nested in a clade that includes pink-spored, saprotrophic and pink-spored ECM *Entoloma* species as well as the white-spored saprotrophic species *Clitocybe hesleri,* but this group lacks statistical support. Macroscopic photograph (B) shows fresh orange, tricholomatoid mushrooms of Agaricales TH9235 (Bar = 10 mm). Close-up photograph (C) illustrates a large cluster of ECM *Dicymbe* roots colonized by the white mycelium of Agaricales TH9235 (Bar = 10 mm).

The sporocarp ITS rDNA sequence from Agaricales TH9235 was identical to ITS rDNA sequences from ECM roots of *D. jenmanii*, confirming the ECM status of this fungus (Fig. 4). While most of the ECM fungal taxa in Guyana can be identified to genus or species by morphology and/or sequence homology in the ITS and 28s rDNA, this is not the case for Agaricales TH9235. Although the fungus superficially resembles species in the genus *Tricholoma* (Agaricales, Basidiomycota), a genus of terrestrial white-spored mushrooms that form ectomycorrhizas, blastN searches based on ITS rDNA indicated that this species is not closely related to *Tricholoma* and shares no obvious homology in the ITS1 or ITS2 regions with any *Tricholoma* taxa in GenBank. BlastN matches of the 5.8s region were inconsistent and provide only low matches to named sequences of Agaricales, suggesting that this mushroom may represent a unique lineage within the order (e.g. a previously unknown genus or family). The equivocal phylogenetic relationships of Agaricales TH9235 were corroborated by inconclusive blastN results from three other gene regions (mtLSU, 28S rDNA and 18S rDNA) that are more conserved than the ITS1 and ITS2 spacer regions (Table 3). For example, blastN results based on 28s rDNA suggested affinities with pink-spored agaricoid fungi in the genera *Entoloma* and *Claudopus*, 18S blastN results suggest possible relationships with white-spored taxa in the genera *Clitocybe* or *Hydropus*, and mtLSU blastN results suggest possible relationships with either the white-spored genus *Amanita* or the pink-spored genera *Pluteus* and *Volvariella* (Table 3). Some of these genera are known to form ECM associations (e.g. *Amanita*, some *Entoloma*) whereas others are considered saprotrophic (e.g. *Clitocybe, Pluteus*) [36].

**Table 3:** Affinities of Agaricales TH9235 based on BlastN analysis of three gene regions (18S rDNA, 28S rDNA, mtLSU).

18S rDNA (GenBank # KC162210)	Trophic status	Spore color	Number of shared nucleotides	Percent similarity
*Clitocybe* aff. *fellea* (HQ728535)	saprotrophic	white	708/741	96%
*Hydropus marginellus* (DQ444856)	saprotrophic	white	709/741	96%
**28S rDNA (GenBank # KC162209)**				
*Entoloma tectonicola* (GQ289196)	saprotrophic	pink	476/530	90%
*Claudopus rupestris* (HQ731515)	saprotrophic	pink	472/528	89%
*Entoloma griseolazulinum* (GQ289166)	ectomycorrhizal	pink	476/535	89%
**mtLSU DNA (GenBank # KC162208)**				
*Amanita pudica* (HQ540041)	ectomycorrhizal	white	287/295	97%
*Volvariella volvacea* (HQ540077)	saprotrophic	white	287/296	97%
*Pluteus petasatus* (HQ540076)	saprotrophic	pink	287/296	97%

BlastN results based on ITS rDNA are not shown because they are uninformative (see text). In addition to the number of shared nucleotides and the percent similarity shared between Agaricales TH9235 and each of the top BLAST hits, the trophic mode and spore color of each species is also shown.

Efforts to shed light on this enigmatic fungus through phylogenetic analysis based on 28S rDNA were inconclusive. Phylogenies produced by both maximum likelihood (Fig. 4) and maximum parsimony (data not shown) suggest that Agaricales TH9235 may be related to species of *Entoloma* and *Clitocybe,* although there is no statistical support for this relationship. The mushrooms that clustered close to TH9235 include pink-spored and white-spored taxa and include both putatively ECM fungi (e.g. *Entoloma griseolazulinum* from subgenus *Entoloma*) as well as saprotrophs (*Clitocybe hesleri*, *Entoloma tectonicola* in subgenus *Inocephalus* and *Entoloma areneosum* in subgenus *Pouzarella*) [42].

## Discussion

This is the first in-depth molecular study of ECM fungi associated with *P. dipterocarpacea* spp. *dipterocarpacea*. Given the moderate level of sampling and the random selection of ECM roots for molecular analysis, we detected a relatively high ECM species richness on this unusual host plant (40 OTUs) and in general for this tropical savanna-forest mosaic (52 OTUs). Moyersoen’s previous studies [11], [24] that examined the ECM fungal community associated with *P. dipterocarpacea* spp. *nitidum* in Venezuela sequenced rDNA from ECM roots and sporocarps to provide evidence of ten ECM fungal lineages (/amanita,/boletus,/cantharellus-craterellus,/clavulina,/coltricia,/cortinarius,/hydnum,/inocybe,/russula-lactarius,/sebacina, and/tomentella-thelephora). We discovered two additional well-established ECM lineages previously unknown on the roots of *P. dipterocarpacea* (/elaphomyces and/hysterangium) as well as the presence of two putatively new lineages (/polyporales1 and/agaricalesTH9235) (see below). Given that our sampling of *P. dipterocarpacea* fungi remained far below saturation (Fig. 4), the cumulative data suggest that both subspecies of *P. dipterocarpacea* probably associate with a wide diversity of ECM fungi over their range in Guyana and Venezuela [24]. At the lineage level, the diversity of ECM fungi associated with *P. dipterocarpacea* is similar to what has been documented with dipterocarps in Southeast Asia and Africa [8], [17], [43].

Despite the high diversity of ECM fungi documented on the roots of *P. dipterocarpacea*, we did not find evidence of strong host effects or evidence that the ECM fungal community in this savanna was dramatically different from nearby *Dicymbe*-dominated rainforest ECM fungal communities. *Pakaraimaea dipterocarpacea* and *D. jenmanii* represent two distantly related plant lineages within the angiosperms that have separately evolved the ability to form the ECM symbiosis [44]. Although both plants belong to the rosid radiation within the eudicots, *Pakaraimaea* belongs to subclass Malvidae whereas *Dicymbe* belongs to subclass Fabidae [45]. Despite the phylogenetic distance of the host plants, we found that most of the common ECM species were multi-host generalists that were detected on the roots of both hosts (Fig. 2). Also, many of these taxa are present at nearby rainforest sites where *P. dipterocarpacea* is absent [9], [25], a fact reinforced by the overlap of many taxa as sporocarps [25].

Despite this low level of fungal specialization, it is notable that four common ECM fungi preferred either *P. dipterocarpacea* or *D. jenmanii* (Fig. 2). Nonetheless, this level of host preference is low when compared with levels found in some other tropical ecosystems and many temperate ecosystems with multiple sympatric hosts, where species of ECM fungi may exhibit strong preference for one host plant lineage over another [10], [46], [47]. The level of host preference in this study, however, was actually more pronounced than in the larger study in nearby rainforest. In that study we examined three leguminous host species, sampled almost four times as many ECM roots, and recovered 118 ECM fungal species but found evidence of host preference in only one fungal species [9]. Two other recent studies of ECM trees in lowland tropical forests in Africa have also reported low levels of ECM host preferences and a high degree of mycobiont sharing among locally sympatric plants in the Fabaceae, Dipterocarpaceae, and Phyllanthaceae [43], [48]. A similar phenomenon of extensive host-sharing has also been found in some, but certainly not all, temperate forest ecosystems [49].

The high degree of host sharing may also partially explain why the ECM fungal community in this tropical savanna ecosystem was compositionally similar to that of closed-canopy rainforests of the region. Due to the highly oligotrophic white sand soils, the higher fire frequency, and the presence of a non-leguminous host lineage (e.g. Dipterocarpaceae), we had expected to find a distinct ECM community. However, 67% of the 52 OTUs that we documented on roots at the Pegaima site as well as 56 out of 82 (68.3%) of the ECM sporocarp species had been previously documented at one or more Fabaceae-dominated rainforest sites in the nearby Upper Potaro Basin, suggesting a fairly homogenous pool of regional ECM fungi. This pattern contrasts with many temperate zone forests where ECM fungal communities often exhibit marked spatial autocorrelation and the dominant ECM species can vary significantly in nearby stands of trees [7], [50]. Furthermore, the importance of edaphic factors in shaping tropical ectomycorrhizal communities has been suggested by studies of paleotropical dipterocarps in Borneo [8], [51].

While many ECM fungi are shared by multiple host plant lineages across sites in Guyana, it was notable that in the present study almost all of the dominant ECM fungal lineages either form dense clusters of ECM roots (e.g./russula-lactarius) or produce ectomycorrhizas equipped with extensive extramatrical hyphal cords for medium- to long-distance soil exploration (e.g./boletus and/cortinarius) [52]. In the present study the ECM fungal lineages that were notably less diverse and dominant on ECM roots as compared to the Smith *et al.* [9] rainforest study were those which exhibited short-distance exploration strategies with minimal hyphal cord development (e.g./clavulina,/sebacina, and/cantharellus). One explanation for the abundance of ECM fungi with long-distance ECM exploration types and/or large ECM clusters may be that they are adapted to the physico-chemical aspects of the nutrient poor white sand savanna soils or to respond favorably to fire disturbance. Alternatively, it may be that our rapid ECM sampling procedures, which included less root sorting and less intense sampling, were more likely to detect these more robust and noticeable ECM types.

Although most of the ECM types that we documented belonged to fungal lineages known from other parts of the world, one unusual ECM type had the same ITS rDNA sequence as large, orange, tricholomatoid mushrooms found fruiting directly on soil under *D. jenmanii* and *P. dipterocarpacea*. This ECM fungus is a member of the Tricholomatoid clade of the Agaricales [53] but is highly divergent compared to any other known species (Table 3, Fig. 4). Sequences obtained from Agaricales TH9235 suggest that the fungus cannot be convincingly placed within any of the four independently derived ECM lineages within the Tricholomatoid clade (i.e./entoloma,/paralyophyllum,/catathelasma, and/tricholoma) [36]. Agaricales TH9235 may therefore represent a unique evolutionary branch within the order that independently evolved the ability to form ECM in the Neotropics. If this is true, it would be the first documented case of a tropical-endemic ECM fungal group. More robust phylogenetic analyses are necessary to address this hypothesis in the future.

Whether based on the number of species detected per tree or the results of diversity indices, the ECM fungal species diversity indicated by root-based sampling in the Pegaima ecosystem appears lower than that of a nearby rainforest dominated by leguminous trees [9]. This result may be due in part to the comparatively lower sampling intensity, minimal morphological sorting of mycorrhizas, and lower success rate in PCR amplification in the present study. Smith *et al.* [9] achieved ca. 90 percent sequencing success when roots were stored directly in CTAB extraction buffer compared to ca. 80 percent success rate in this study where roots were rapidly air-dried with silica gel. Methodological issues aside, the comparison of sampling effort curves from the two different studies suggests that a more complete sampling of this savanna study site would yield similar diversity to nearby rainforest sites (Fig. 3). Additionally, the 86 sporocarp species of ECM fungi collected from the Pegaima site during two short expeditions totals nearly half of the 174 species recovered from the Potaro rainforest site over a 10 year sampling period, suggesting that many more sporocarp species remain to be discovered at Pegaima [25].

A relatively small number of tropical ECM communities have thus far been studied using molecular techniques but the available data suggest that tropical ecosystems are highly variable in terms of both ECM fungal diversity and the level of ECM host preferences. Ecosystems inhabited by ECM hosts that are large, dominant trees growing close together appear to have relatively high ECM fungal diversity but often low levels of fungal host preferences [7], [9], [43], [48]. In contrast, tropical forests with phylogenetically diverse ECM host plants occurring at low densities in otherwise arbuscular mycorrhizal dominated plant communities have low ECM fungal diversity but often have mycobionts that exhibit distinct host preferences [10]. Thus it appears that several factors, including size of the host plants, host distribution or dominance within the community, and host phylogenetic relationships, may all be important in governing ECM fungal diversity and host associations in tropical habitats. Certainly more studies of tropical ECM plants and their associated ECM fungal communities are warranted to further explore these intriguing patterns.
